# Biorefinery process for protein extraction from oriental mustard (*Brassica juncea *(L.) Czern.) using ethanol stillage

**DOI:** 10.1186/2191-0855-2-5

**Published:** 2012-01-12

**Authors:** Kornsulee Ratanapariyanuch, Robert T Tyler, Youn Young Shim, Martin JT Reaney

**Affiliations:** 1 Department of Food and Bioproduct Sciences, University of Saskatchewan, 51 Campus Drive, Saskatoon, SK S7N 5A8, Canada; 2 Department of Plant Sciences, University of Saskatchewan, 51 Campus Drive, Saskatoon, SK S7N 5A8, Canada

**Keywords:** Biorefinery, Protein extraction, Thin stillage Mustard, Salt concentration, Ethanol

## Abstract

Large volumes of treated process water are required for protein extraction. Evaporation of this water contributes greatly to the energy consumed in enriching protein products. Thin stillage remaining from ethanol production is available in large volumes and may be suitable for extracting protein rich materials. In this work protein was extracted from ground defatted oriental mustard (*Brassica juncea *(L.) Czern.) meal using thin stillage. Protein extraction efficiency was studied at pHs between 7.6 and 10.4 and salt concentrations between 3.4 × 10^-2 ^and 1.2 M. The optimum extraction efficiency was pH 10.0 and 1.0 M NaCl. Napin and cruciferin were the most prevalent proteins in the isolate. The isolate exhibited high *in vitro *digestibility (74.9 ± 0.80%) and lysine content (5.2 ± 0.2 g/100 g of protein). No differences in the efficiency of extraction, SDS-PAGE profile, digestibility, lysine availability, or amino acid composition were observed between protein extracted with thin stillage and that extracted with NaCl solution. The use of thin stillage, in lieu of water, for protein extraction would decrease the energy requirements and waste disposal costs of the protein isolation and biofuel production processes.

## Introduction

*Brassica *spp. oilseeds are grown throughout the world as sources of vegetable oil and protein-rich animal feed (Henriksen et al. 2009). According to statistical data from the Canada Grains Council (2011), the average annual production of Canadian canola over the period 2001-2010 was 9.2 million tonnes, and the Canadian oilseed crushing industry produced an average of 2.1 million tonnes of canola meal annually between 2001-2010. Commercial oilseed extraction may include solvent extraction, mechanical expeller-press extraction, or combinations of mechanical and solvent extraction to produce oil and meal. Canola meal is the portion remaining after extraction of oil from canola seed and it is widely used as a protein source in poultry, swine, beef, and dairy cattle feeds because of its excellent amino acid profile (Hickling 2011).

Thin stillage (TS) is a dilute stream of organic and inorganic compounds produced as a coproduct of the ethanol industry. Usually, TS is processed by drying than added to distillers dried grains (DDG) to produce DDG with solubles (DDGS). The latter is used in animal feeds. In the manufacture of DDGS, TS is first concentrated into syrup before mixing with wet distillers grains. TS drying consumes about 40-45% of the thermal energy and 30-40% of the electrical energy utilized in a dry-grind facility (Wilkins et al. 2006). The energy required to evaporate the large amount of water entrained in TS is a major cost in the ethanol industry and contributes to the poor lifecycle assessment of ethanol production (Bremer et al. 2010). To overcome the losses in energy for this process several strategies have been proposed including feeding wet distiller's grains with solubles. This has the advantage of decreasing the cost of drying but necessitates transporting water with the feed product to the animals. In addition the wet products may not be suited for storage.

Production of protein isolates is equally inefficient. For examples, Newkirk et al. (2006) disclose a multistage protein extraction and recovery process using water and CaO to adjust pH; Diosady et al. (1989) extracted 100 g of rapeseed meal with 1,800 g of water; and Murray (1998) extracted 50 kg of commercial canola meal with 500 L of water. In all of these extractions the percent of protein concentrate recovered to water used in extraction and processing is less than 3%. Therefore, the consumption of large volumes of water, and its subsequent remediation are costly barriers to the economic production of protein concentrates and isolates.

If the ethanol, oilseed, and protein processing plants are in close physically proximity, TS from the ethanol production plant could be used directly as process water by the protein processing facility. The ethanol producer would avoid the costs of evaporating and drying or treating TS. The protein producer would not have to purchase water for the process and would reduce the energy costs to heat the water for protein extraction. The oilseed processor would provide defatted meal as raw material for protein extraction, and in the case of an oilseed plant that also produces biodiesel, alkaline glycerol, a byproduct from biodiesel plants, could be used for pH adjustment in the protein extraction process. Thus, the ethanol, biodiesel and protein processes would benefit.

In a previous study (Ratanapariyanuch et al. 2011), we thoroughly characterized TS to determine the presence of compounds that might affect protein extraction. The use of TS for protein extraction from canola or mustard meal has not been reported previously. However, as described above, the use of TS might offer several advantages in the extraction of protein from oilseed meal.

## Materials and methods

### Materials, chemicals and reagents

Oriental mustard seed cultivar (*B. juncea *(L.) Czern. cv. AC Vulcan) seed was obtained from Agriculture and Agri-Food Canada, Saskatoon Research Centre (Saskatoon, SK, Canada). All seed was from the 2006 harvest and was grown on plots near Saskatoon. Pound-Maker Agventures Ltd. (Lanigan, SK, Canada) provided TS from wheat. Samples of TS were stored at 4°C for up to 4 months until used. TS samples were centrifuged at 1050 × *g *for 20 min at 4°C (Model Avanti^® ^J-E, Beckman Coulter Canada Inc., Mississauga, ON, Canada). Glycerol containing approximately 10% KOH was provided by an industrial biodiesel processor (Milligan Biotechnology Inc., Foam Lake, SK, Canada). Reagents and chemicals, unless otherwise noted, were purchased from Sigma-Aldrich (St. Louis, MO, USA).

### Defatted meal preparation

Mustard seed was extracted mechanically using a continuous screw expeller (Komet, Type CA59 C; IBG Monforts Oekotec GmbH & Co. KG, Mönchengladbach, Germany) with a 6 mm choke and operating with a screw speed of 93 rpm. Oil remaining in the press-cake was removed using hexane as a solvent (Milanova et al. 2006; Oomah et al. 2006) and the residual hexane in the defatted meal was removed in a fume hood overnight.

### Protein content

Protein content of mustard seed and fractions were determined by the Kjeldahl method as modified by AOAC method 981.10 (AOAC 1990). Mustard seed and defatted meal samples (0.5 g) were digested by heating with concentrated H_2_SO_4 _in a heating/digestion block using a package of Kjeldahl digestion mixture 200 (VWR Scientific, Mississauga, ON, Canada) as a catalyst. After digestion, samples were distilled using a steam distillation unit (Büchi Analytical Inc., New Castle, DE, USA) with 30% (w/v) NaOH. Boric acid (4%) was used to trap ammonia from the distillation. The distillate was titrated with 0.2 N HCl using an N-Point indicator (Titristar N point indicator, EMD Chemicals Inc., Gibbstown, NJ, USA). Nitrogen concentration (N in %) was used to estimate protein concentration (%) by means of a nitrogen-to-protein conversion factor 5.7 (Sosulski et al. 1990) for TS and 5.5 (Lindeboom and Wanasundara 2007) for mustard seed, meal, and protein.

### Oil content

The oil content was determined using a Goldfisch Extractor (Model 22166B, Labconco Corp., Kansas City, MO, USA) according to AOAC method 960.39 (AOAC 1990). Samples (20 g) were ground for 30 s in a coffee grinder to pass through a 1 mm screen. A portion of the ground sample (3 g) was weighed on a filter paper (Whatman No. 4), which was then folded. The samples were placed in cellulose thimbles (25 mm × 80 mm, Ahlstrom AT, Holly Spring, PA, USA) and extracted for 6 h with hexane (50 ml). The hexane was distilled from the oil extraction beakers, after which the beakers were heated at low temperatures (30-40°C) using a hot plate placed in a fume hood. The beakers were then transferred to an oven (105°C) for 30 min and then allowed to cool to room temperature (25°C) in a desiccator.

### Moisture content

The moisture content of mustard seed and defatted meal was determined according to AOAC method 950.46 (AOAC 1990) using a Mettler Toledo halogen moisture analyzer (Model HB43, Columbus, OH, USA), which employed a quartz heater to dry samples of material (1.0 g) at 105°C until the mass varied less than ± 0.001 g over a 30 s. The samples were allowed to cool to room temperature in a desiccator for at least 1 h before weighing. Selected samples were frozen at -20°C and lyophilized for 48 h.

### The effects of pH and salt on protein extraction

The amount of liquid used for protein extraction may determine both extraction efficiency and economics. A 1:30 ratio of defatted meal to solvent, and an extraction time of 120 min were utilized in this study, as recommended by Diosady et al. (2005). To avoid protein precipitation and achieve the maximum protein extraction, it is important to avoid pH near the isoelectric point of protein. Based on the literature, the isoelectric precipitation of *B. juncea *protein has been found to occur at approximately pH 6.0 (Moure et al. 2006). Therefore, alkaline conditions (pH > 7.0) were chosen to study protein extraction. Ground defatted meal (5.0 g) was mixed with 150 ml of centrifuged TS. The pH of the system was adjusted to pH 7.6-10.4 using alkaline glycerol from a biodiesel plant (~10% KOH) or 1.0 N HCl. NaCl was used to adjust the ionic strength of the centrifuged TS. The concentrations of NaCl ranged from 3.4 × 10^-2 ^M to 1.2 M. The pH and salt concentrations employed are provided in Table 1.

**Table 1:** Coded values of independent variables used to study the effect of pH and salt (NaCl) concentration on protein extraction efficiency

Independent variable	Code level*^a^*				
	
	-1.414	-1.0	0.0	1.0	1.414
pH	7.6	8.0	9.0	10.0	10.4
Salt concentration (M)	0.034	0.2	0.6	1.0	1.2

*^a ^*All levels of each factor were chosen based on a central composite rotatable design.

The mustard meal-TS mixture was stirred continuously for 2 h at room temperature (25°C). After stirring, the solution was centrifuged at 10,000 rpm for 10 min at 4°C to remove suspended solids. The supernatant was freeze-dried, after which the protein content of the freeze-dried protein of the undissolved solids were analyzed. The moisture content of the undissolved solids was also determined. The conditions that provided the maximum protein extraction efficiency in this study (NaCl concentration of 1.0 M and pH 10.0) were used in subsequent studies of the effects of TS constituents on protein extraction efficiency. A control extraction with an alkaline NaCl solution (1.0 M NaCl in deionized water, pH 10.0), hereafter termed NaCl solution, was conducted. The quality of the protein products from the control and TS extractions was compared.

### Thin stillage composition

The composition of TS was characterized according to Ratanapariyanuch et al. (2011). Nuclear magnetic resonance and high-performance liquid chromatography (HPLC) were utilized to determine the content of organic compounds including ion chromatography and inductively coupled plasma mass spectroscopy (ICP-MS) provided a detailed analysis of inorganic constituents.

### Protein extraction efficiency

Protein was removed from TS *via *ultrafiltration prior to its use for protein extraction from mustard meal. Centrifuged TS was filtered through a 3,000 MWCO regenerated cellulose membrane (Millipore Corp., Bedford, MA, USA) using a stirred ultrafiltration cell (Millipore Corp., Bedford, MA, USA), running at 55 psi with a shear rate of 200 rpm. A solution of NaCl (1.0 M and a pH of 10.0) was selected to obtain the highest protein extraction efficiency (based on results from the previous experiment above). Protein was extracted as described above. The supernatant from the centrifuged protein solution was dialyzed using Spectra/Por molecular-porous membrane tubing (3,500 MWCO, Spectrum Laboratories Inc., Rancho Dominguez, CA, USA) at a supernatant to deionized distilled water ratio of 1:1,000. Water exchange with fresh deionised water was repeated three times a day until the conductivity of permeate water was equal to that of deionised distilled water after 8 h of dialysis. The protein solution obtained by dialysis was freeze-dried. Freeze-dried protein and undissolved solids were analyzed for protein content, and the moisture content of undissolved solids was also determined. Protein products from TS and NaCl extraction were pooled according to extraction solution type, and then analyzed to determine the molecular weight, peptide sequence, amino acid composition, digestibility, and lysine availability of the proteins.

### Molecular weight

Molecular weights of the extracted proteins were determined by electrophoresis separation using sodium dodecyl sulfate-polyacrylamide gel electrophoresis (SDS-PAGE) (Laemmli, 1970). Ten micrograms of protein from TS or NaCl extraction and 5.0 μg of SeeBlue^® ^Plus2 Pre-Stained Standard (Invitrogen, Carlsbad, CA, USA) with a range of 4**-**250 kDa were applied onto 8.6 cm × 6.8 cm Ready Gels (Tris-HCl 4**-**15%, 10 wells, Bio-Rad Laboratories, Hercules, CA, USA). Each of the proteins products was mixed at a 1:1 ratio with loading buffer (1.0 M Tris-HCl, pH 6.8, containing 20% glycerol, 10% SDS, 0.4% bromophenol blue), and heated on a Gene Amp PCR System 9700 (Applied Biosystems, Foster City, CA, USA) at 95°C for 5 min. The Mini-PROTEAN 3 cell (Bio-Rad Laboratories, Hercules, CA, USA) was filled with running buffer (Tris base 3.028 g/l, glycine 14.414 g/l, SDS 1.0 g/l) adjusted pH to 8.3, and electrophoresis was performed for 30 min at 50 V. The voltage was then increased to 100 V for 70 min. After electrophoresis, the gels were stained with 0.1% of Coomassie Brilliant Blue R-250 (Sigma, St. Louis, MO, USA) mixed with 40% methanol and 10% acetic acid. Subsequently the stained gels were destained with 40% methanol and 10% acetic acid to remove the background.

### Peptide mass fingerprinting

Each of the stained protein containing bands observed in the electrophoresis gel was excised from reference gels for identification by matrix-assisted laser desorption ionization time-of-flight mass spectrometry (MALDI-TOF MS) according to the method of Aluko et al. (2004). After MALDI-TOF analysis of tryptic peptides, mass tags were searched against the mustard UniGene database using the MS-FIT program of Protein Prospector (University of California, San Francisco, CA, USA) with autocatalytic trypsin fragments as internal calibration standards. All searches were performed against the National Center for Biotechnology Information (NCBI) mustard UniGene database.

### Amino acid composition

The amino acid profiles of extracted proteins were determined using the method of Llames and Fontaine (1994). Performic acid and HCl were used to oxidize and hydrolyze the proteins, respectively. Hydrolysates were analyzed for amino acids using an analytical ion exchange column (AA911, Transgenomics Inc., Omaha, NE, USA) and post column derivitization with *ortho*-phthaldialdehyde (OPA). An Agilent 1100 series HPLC system (Agilent Technologies, Waldbronn, Germany) and fluorescence detector (RF-551, Shimadzu Scientific Instruments, Columbia, MD, USA) were employed. Amino acids were quantified with the internal standard method of measuring the absorption of reaction products with ninhydrin at 570 nm. Tryptophan was determined by HPLC with fluorescence detection (extinction 280 nm, emission 356 nm) after alkaline hydrolysis with barium hydroxide octahydrate for 20 h at 110°C (European Commission 2000). Tyrosine was not determined. Supplemented amino acid was determined by extraction with 0.1 N HCl (European Commission 1998).

### *In vitro *digestibility

The digestibility of extracted protein was determined using the multi-enzyme technique of Hsu et al. (1977). Lyophilized protein samples extracted with TS and NaCl solution were dissolved in deionized water (6.25 mg protein/ml). The protein solutions (25 ml) were adjusted to pH 8.0 with 0.1 N HCl or NaOH, while stirring at 37°C in a water bath. The multi-enzyme solution (1.6 mg/ml trypsin, 3.1 mg/ml chymotrypsin, and 1.3 mg/ml peptidase) was prepared in water adjusted to pH 8.0 and stored in an ice bath. Digestions were conducted by adding the multi-enzyme solution (2.5 ml) to 25 ml of protein solution while stirring at 37°C. The pH of the protein solution was recorded over a 10 min period using a recording pH meter. The percent protein digestibility was calculated by the following eq 1:

(1) Digestibility(%)=210.46-18.10X

X is the pH at 10 min. The enzyme blank was run in 0.001 M phosphate buffer, pH 8.0.

### Lysine availability

Lysine availability of extracted protein was measured using a fluorometric technique (Ferrer et al. 2003). A reconstituted protein sample (50 μl) containing 0.3-1.5 mg of protein was mixed with deionized water (950 μl), and then 1 ml of SDS solution (120 g/l) was added. An OPA solution was prepared by combining 80 mg OPA in 2 ml 100% ethanol, 50 ml sodium tetraborate buffer (pH 9.7-10.0), 5 ml SDS (200 g/l), and 0.2 ml β-mercaptoethanol. OPA solution (3 ml) was added to 100 μl of the reconstituted protein solution. The mixture was incubated for 2 min at 25°C while shaking. Fluorescence was measured between 2 and 25 min at 455 nm (Pi-Star 180 CD spectrophotometer, Applied Photophysics Ltd., Leatherhead, U.K.). The absorbance value of the protein sample was corrected by the absorbance of a blank and the absorbance of the interference. The blank mixture (1 ml of SDS solution, 120 g/l, and 1 ml of deionized distilled water) was incubated at 4°C for 12 h, after which it was sonicated (Branson 3200R-1, Sonicator, Branson Cleaning Equipment Company, Danbury, CT, USA) for 15 min at 25°C. Interference in the determination stems from small peptides, free amino acids, and amines. In order to determine the interference, trichloroacetic acid (TCA) was added to precipitate protein in the sample solution (2 ml of 10% (w/v) TCA and 2 ml of protein extract), which was then centrifuged at 827*g *(Allegra X-22R Centrifuge, Beckman Coulter Canada Inc., Mississauga, ON, Canada). Blank controls were prepared by combining 900 μl of deionized water, 1 ml of SDS solution (120 g/l), and 100 μl of supernatant. A calibration curve was prepared using a mixture of casein from bovine milk at concentrations ranging from 0.1 to 2.0 mg/ml (lysine contents of 8.48 × 10^-3 ^to 0.169 mg lysine/ml) dissolved in 0.1 M sodium tetraborate buffer (pH 9.0).

### Color

The color of extracted protein was determined using a HunterLab system (Color Flex, Hunter Associates Laboratory Inc., Reston, VA, USA). The illuminator condition was set at D65 (daylight), and the observer at 10°. In the Hunter scale, '*L*' measures lightness and varies from 100 for white to zero for black. The chromaticity value '*a*' measures redness when positive, gray when zero, and greenness when negative. The '*b*' value measures yellowness when positive, gray when zero, and blueness when negative. The colorimeter was calibrated with standard black and white calibration tiles provided with the instrument before measuring the colors of TS and NaCl solution.

### Large-scale protein extraction

The ratio of ground defatted meal to TS was increased to 1:5 to simulate a more practical industrial process. Ground defatted meal (180 g) was mixed with centrifuged TS (900 ml) having a NaCl concentration of 1.0 M. The pH was adjusted and protein extracted as described previously. The supernatant from protein extract centrifugation was dialyzed using Spectra/Por molecular porous membrane tubing (Spectrum Laboratories Inc., Rancho Dominguez, CA, USA), 6,000-8,000 MWCO, at a ratio of 1:20 supernatant to deionized water. The meal was extracted twice more with 900 ml of centrifuged TS for 2 h per extraction (1:5, meal: centrifuged stillage ratio). The supernatant from each extraction was dialyzed as described above. Water exchange with fresh deionized water was repeated until the conductivity of the permeate water was equal to that of deionized water after 8 h of dialysis. The three dialyzed protein extracts were combined and sub-sampled, and the sub-samples were lyophilized and subsequently analyzed for protein content.

### Comparison of protein extraction efficiency with that of a published protocol

Using the protocol of Milanova et al. (2006), ground defatted meal (20 g) was mixed with 200 ml of 0.6 M NaCl solution. The pH of the mixture was adjusted to 6.8 with a 0.1 N HCl solution, stirred continuously for 30 min at 25°C and then centrifuged at 10,000 rpm for 10 min. The supernatant was filtered using a stirred cell with a 3,000 MWCO membrane until the volume of protein solution was 10 ml. Subsequently, the protein solution was diafiltered (3,000 MWCO) using 500 ml of 0.6 M NaCl solution at pH 6.0, until the volume of the solution was 20 ml. The concentrated protein and salt solution (20 ml) was then diluted 15-fold (to 300 ml) with chilled water (4°C) to form a discrete protein (micelle) in the aqueous phase. The protein micelle was allowed to settle to form an amorphous, gelatinous mass. The protein mass was centrifuged at 10,000 rpm for 10 min to separate protein particles from the liquid. The protein sediment was lyophilized and subsequently analyzed for nitrogen content using the Kjeldahl method.

### Statistical analysis

All measurements were undertaken in triplicate. The efficiency of protein extraction was determined using a response surface methodology (RSM). Five levels of each factor (pH and salt concentration) were chosen based on a central composite rotatable design (CCRD) (Table 1) (Kuehl 2000).

## Results

### Composition of *B. juncea *mustard seed and defatted meal

The protein, oil, and moisture contents of whole mustard seed were 22.1 ± 0.1, 38.7 ± 0.2, and 4.8 ± 0.1%, respectively. For defatted meal, the protein, oil, and moisture contents were 32.3 ± 0.2, 4.1 ± 0.1, and 6.3 ± 0.1%, respectively.

### The effect of pH and salt on protein extraction

Both salt concentration and pH affect protein solubility. The effect of these two variables on the efficiency of protein extraction was studied in order to determine the optimum conditions for protein extraction from mustard meal. Maximum protein extraction efficiency was achieved at the highest pH and NaCl concentration employed (10.4 and 1.2 M, respectively) (Table 1).

### Protein extraction using thin stillage and sodium chloride solution

#### Efficiency of protein extraction

**Figure 1:**
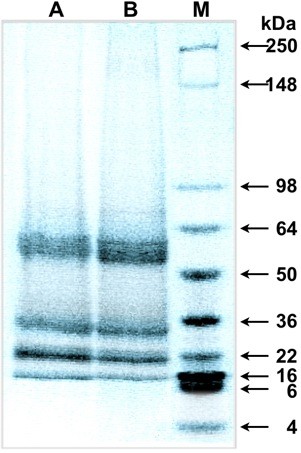
**SDS-PAGE separation of protein extracted by different methods**. Lane A, thin stillage; lane B, NaCl solution; lane M, broad range molecular marker.

The efficiency of protein extraction may be affected by the presence of compounds, such as divalent cations, which are found in industrial TS (Ratanapariyanuch et al. 2011) but not in the NaCl solution. TS used in this study was first filtered with an ultrafiltration membrane of 3,000 MWCO to remove large molecules such as proteins and polysaccharides. The conditions that produced the highest protein extraction efficiency (pH 10.0, NaCl concentration of 1.0 M) in preliminary experiments were employed. The results did not show any significant differences in the efficiency of protein extraction obtained using TS (60.1 ± 4.4%) or NaCl solution (56.3 ± 4.1%). The molecular weights of the proteins extracted by TS and NaCl solution were determined by SDS-PAGE to be 14, 18-20, 20-22, 34, and 55 kDa (Figure 1).

#### Protein identification

The masses and peptide mass fingerprint of the peptides are presented in Table 2. A number of abundant peaks from singly-charged tryptic peptides ranging from 973.49 to 2,088.19 m/z were observed. The results showed that for napin, only a 14 kDa peptide fragment was observed. The masses obtained from the tryptic digests matched predicted digestion products. Specifically predicted tryptic digestion fragments [12 to 20 (EFQQAQHLR) and 100 to 109 (IYQTATHLPR)] were matched with the sequence of *B. juncea *1-E in the database (Monsalve et al. 1993). Peptide fragments with masses of 18-20, 20-22, 34, and 55 kDa were observed that matched the predicted masses from tryptic digestion of cruciferin. The protein peak appeared to consist of a single protein purified to near-homogeneity as indicated by both the MALDI-TOF MS data and SDS-PAGE analysis (Table 2 and Figure 1).

**Table 2:** Amino acid sequences of tryptic peptide fragments of protein extracted from *B. juncea *using thin stillage

Subunit mass (kDa)	Fragment sequence	Calculated mass (m/z)	Actual mass (m/z)	Sequence assignment	Position
14	EFQQAQQHLR	1155.58	1156.68	Allergen *B. juncea *1-E	12-20
	IYQTATHLPR	1198.64	1199.75	Allergen *B. juncea *1-E	100-109
18-20	GLPLEVISNGYQISPQEAR	2070.07	2071.21	Cruciferin	338-386
20-22	GLPLEVISNGYQISLEEAR	2087.09	2088.19	Cruciferin	66-84
	GLPLEVISNGYQISPQEAR	2070.07	2071.19	Cruciferin	368-386
34	CSGFAFER	972.41	973.49	Cruciferin	62-69
	VQGQFGVIRPPLR	1465.85	1466.94	Cruciferin	251-263
	IEVWDHHAPQLR	1499.76	1500.83	Cruciferin	50-61
55	GPFQVVRPPLR	1264.74	1265.79	Cruciferin	288-298
	VQGQFGVIRPPLR	1465.85	1466.91	Cruciferin	251-263
	IEVWDHHAPQLR	1499.76	1500.81	Cruciferin	50-61
	GLPLEVISNGYQISPQEAR	2070.07	2071.13	Cruciferin	420-438

#### Amino acid composition

The amino acid composition of protein extracted from mustard meal using TS and NaCl solution was analyzed by HPLC (Table 3). The differences in amino acid content among proteins extracted with TS and NaCl solution were slight. The standard deviation of the valine content of protein extracted with NaCl solution was high, as the baseline of the HPLC chromatogram was not smooth. In proteins extracted by each of the two solutions, glutamic acid and methionine were present in the highest and lowest concentrations, respectively. Of the essential amino acids, leucine, and methionine were present in the highest and lowest concentrations, respectively.

**Table 3:** Amino acid composition of protein extracted from *B. juncea *using thin stillage and NaCl solution.

Amino acid	**Thin stillage**^**a**^	**NaCl solution**^**a**^	**Protein isolated from *B. juncea***^**b**^	**FAO**^**c**^
Cysteine	5.3 ± 0.0	5.2 ± 0.2	2.9 ± 0.1	N
Asparagine	5.4 ± 0.2	6.0 ± 0.1	N^d^	N
Methionine	2.2 ± 0.2	2.3 ± 0.0	2.7 ± 0.0	2.5^e^
Threonine	3.1 ± 0.1	3.5 ± 0.2	4.3 ± 0.1	3.4
Serine	3.9 ± 0.1	4.2 ± 0.0	4.5 ± 0.0	N
Glutamic acid^f^	22.2 ± 0.1	23.0 ± 0.2	20.8 ± 0.1	N
Glycine	4.6 ± 0.1	4.9 ± 0.1	5.2 ± 0.0	N
Alanine	3.9 ± 0.1	4.3 ± 0.1	4.4 ± 0.1	N
Valine	6.0 ± 0.2	3.0 ± 0.2	5.2 ± 0.1	3.5
Isoleucine	3.4 ± 0.0	3.8 ± 0.1	3.7 ± 0.1	2.8
Leucine	6.6 ± 0.1	7.5 ± 0.1	7.8 ± 0.0	6.6
Phenylalanine	3.6 ± 0.2	4.1 ± 0.4	4.5 ± 0.0	N
Histidine	4.5 ± 0.1	4.5 ± 0.1	2.8 ± 0.0	1.9
Lysine	5.2 ± 0.2	5.9 ± 0.3	4.9 ± 0.1	5.8
Arginine	7.0 ± 0.2	7.6 ± 0.1	10.0 ± 0.0	N
Tryptophan	N	N	1.5 ± 0.0	N
Tyrosine	N	N	2.3 ± 0.0	6.3^g^
Aspartic acid	N	N	7.0 ± 0.1^h^	N
Proline	N	N	5.6 ± 0.0	N

Data expressed as g/100 g of protein are the mean ± standard deviations (SD) of three analyses.

^a ^1.0 M NaCl added. ^b, c ^From references (Alireza-Sadeghi et al. 2006; FAO 2002).

^d ^N means no analysis.

^e ^Value for methionine + cysteine. ^f ^Value for glutamic acid + glutamine.

^g ^Value for tyrosine + phenylalanine. ^h ^Value for aspartic acid + asparagine.

#### In vitro digestibility

The protein products extracted with TS and NaCl solutions had similar digestibility of 74.9 ± 0.8 and 74.5 ± 0.5%, respectively (Table 4). Alireza-Sadeghi et al. (2006) found that the digestibility of defatted *B. juncea *meal and protein isolated from defatted *B. juncea *meal were 80.6 and 92.4%, respectively. The digestibility of protein from this study was lower than reported by others previously (Table 4).

**Table 4:** *In vitro *digestibility and lysine availability of protein extracted from mustard meal using thin stillage or NaCl solution

Constituent	**Thin stillage**^**a**^	**NaCl solution**^**a**^
Digestibility (%)	74.9 ± 0.8	74.5 ± 0.5
Lysine availability (g/kg of sample)	43.0 ± 0.3	42.0 ± 0.4

Values are the means of triplicate determinations with SD of a single sample.

^a ^1.0 M NaCl added.

#### Lysine availability

The availabilities of lysine from protein product extracted with TS and NaCl solution were similar at 43.0 ± 0.3% and 42.0 ± 0.4%, respectively (Table 4).

#### Color

The color of the protein product extracted with TS (*L *= 56.36 ± 0.08) was darker than that of protein extracted with NaCl solution (*L *= 69.04 ± 0.07) (Table 5). However, *a *(2.34 ± 0.01 - 3.45 ± 0.05) and *b *(19.33 ± 0.01 - 19.55 ± 0.05) values were similar in the two protein products.

**Table 5:** Color of protein extracted from mustard meal using thins or NaCl solution

Color parameter	**Thin stillage**^**a**^	**NaCl solution**^**a**^
*L*	56.36 ± 0.08	69.04 ± 0.07
*a*	3.45 ± 0.05	2.34 ± 0.01
*b*	19.33 ± 0.01	19.55 ± 0.05

Values are the means of triplicate determinations with SD of a single sample.

^a ^1.0 M NaCl added.

## Discussion

The composition of TS was reported separately (Ratanapariyanuch et al. 2011). In brief, stillage contained a number of organic and inorganic constituents that constituted a solution with about 3% dissolved matter. Our original hypothesis was that some of the dissolved constituents might either alter the efficiency of protein extraction or affect the quality of the extracted protein. Neither the efficiency of protein extraction nor the quality of protein was affected by the whole stillage. Therefore, we did not have reported the effect of individual components of the stillage on protein yield and quality. In this study, the relative efficiencies of protein extraction using TS and NaCl solution were used to determine the effect of these solutions. In addition, SDS-PAGE of extracted protein, amino acid sequences of tryptic peptide fragments of extracted protein, digestibility, and lysine availability of extracted protein were compared for protein extracted using TS and NaCl solution.

High pH and salt concentrations are not necessarily practical if they are not cost effective even if they increase protein extraction efficiency. At alkaline pH, most proteins have a net negative charge, which results in strong intramolecular electrostatic repulsion. This would cause swelling and unfolding of protein molecules (Damodaran 1996) and possible loss of functionality. Similarly, when pH is above the isoelectric pH, protein solubility increases. Typically, the maximum solubility of protein occurs in alkaline solutions. Damodaran (1996) noted that when ionic strength is low (NaCl concentration < 0.5 M) the solubility of proteins that contain polar surface domains typically increases. The effects of pH and salt concentration demonstrated in this study are in agreement with the literature. Lindeboom and Wanasundara (2007) extracted protein from yellow mustard (*Sinapis alba*) using water at different pHs (3.5-10.0). They discovered that the protein content of the extracts increased when the pH was above 7.5, and was as high as 25 mg/ml at pH 10.0.

According to the molecular weights of the proteins extracted by TS and NaCl solution (Figure 1), Aluko and McIntosh (2001) reported that a 52 kDa polypeptide was present in a purified 12S globulin storage protein (cruciferin) from *Brassica napus *seed. Aluko et al. (2004) stated that in *S*. *alba *protein isolates, a 2S albumin storage protein (napin) band appeared at 5 kDa and cruciferin bands at 22, 28, and 35 kDa. Aluko and McIntosh (2004) demonstrated that 12 and 13 kDa polypeptides were subunits of the napin of mustard seed. Shim and Wanasundara (2008) reported that a single protein band of 14.5 kDa polypeptides were two polypeptide chains of 4.5 and 10 kDa linked by disulfide bonds. From the above information, it was concluded that the bands found in SDS-PAGE were cruciferin and napin, and that they could be extracted with either TS or NaCl solution. These results were then confirmed by peptide sequencing.

A combination of in-gel trypsin digestion of protein separated by SDS-PAGE followed by MALDI-TOF MS of the digests produced the masses used for searching peptide-mass databases. Table 2 shows the search results, which identifies peptide fragments of *B. juncea*. These results are in agreement with those of Aluko and McIntosh (2001)Aluko et al. (2004)Aluko and McIntosh (2004), and Shim and Wanasundara (2008), as described above. In addition, using fragment exact mass, the same peptide sequences of cruciferin were separated into different bands by gel electrophoresis. This can be explained by: (1) possible degradation of the extracted protein to smaller molecules by enzyme, pH or hydrolysis during processing and (2) the cruciferin present in rapeseed is a member of the 12S globulins which are hexameric molecules consisting of homologous but non-identical subunits (Tandang et al. 2004). Surprisingly, no peptides arising from yeast, bacteria or wheat were found. Only napin and cruciferin were identified in the extracted protein. These proteins isolates are, therefore, similar to those prepared from related *Brassica *species. The potential exists to process these isolates using hydrolytic enzymes to produce bioactive peptides and antioxidants that may be added to feed and food (Xue et al. 2009).

The amino acid composition is comparable to the amino acid composition of proteins isolated from *B. juncea *analyzed by Alireza-Sadeghi et al. (2006). In addition, the quantity of essential amino acids extracted is sufficient to meet Food and Agriculture Organization (FAO) standards (2002) (Table 3). Lysine is frequently the factor limiting the protein quality of mixed diets for human food and animal feed. When the total lysine content was compared with the available lysine content, it was found that approximately 75% of the lysine in the extracted protein would be available in feed. These results agree with those of Larbier et al. (1991) who found that lysine digestibility of whole rapeseed meal, dehulled rapeseed meal, and soybean meal for cockerels were 80.1, 86.0, and 88.9%, respectively. The available lysine values for chicks were 72.8, 78.3, and 85.5%, respectively. The digestibility of the isolates produced in this study was below that reported by Alireza-Sadeghi et al. (2006). The higher reported digestibility may be the result of charcoal adsorption treatment of the mustard protein isolates that was used in that study.

The darker color of protein extracted with TS may be due to the inclusion of colored compounds with the protein or reactions between compounds in the stillage and protein to produce color. In addition, protein extracted with TS could have absorbed colored materials from the alkaline glycerol or TS. Therefore, the compound present in TS may affect the other protein properties such as *in vivo *digestibility, which were not examined in this study. Consequently, other qualities of the extracted protein should be tested in future studies.

The efficiency of protein extraction is affected by the ratio of meal to solvent, where a higher ratio leads to lower efficiencies. However, the energy required to evaporate water from the protein solution in the final processing step would make the overall process inefficient at low meal to solvent ratios. The ratio of ground defatted meal:solvent (1:30, w/v) used in the preliminary experiments would not be practical for industrial application, thus the use of a higher ratio (1:5, w/v) was evaluated. As expected, the results showed that when the meal: solvent ratio used for protein extraction was increased from 1:30 to 1:5, protein extraction efficiency decreased from 80% to 60%. The efficiency of the protein extraction process developed in this study was compared with that of a published protocol (Milanova et al. 2006). In the published protocol, the cold-water treatment caused the protein to salt out in micelle form. The percent recovery from the protein micelle was only 7.6%. This protein recovery was significantly lower than the 80% achieved with extractions at pH 10.0 and a NaCl concentration of 1.0 M. It can be concluded that the process developed in this research was more efficient in terms of protein extraction than the published protocol.

In conclusions, a biorefinery process was developed that linked coproducts of bio-ethanol and biodiesel production. TS was used for protein extraction from defatted *B. juncea *meal, a coproduct of biodiesel production from oilseed. In addition, biodiesel plants can provide alkali to increase pH and protein solubility. Therefore, ethanol, biodiesel, and protein industries benefit from process integration. TS did not affect the efficiency of protein extraction or nutritional qualities of the protein extracts. The use of a byproduct, TS, as a part of a protein extraction process would increase the viability of the linked industrial processes. The current work demonstrates that the protein products of stillage-based extractions are of acceptable quality for use in feeds.

## Competing interests

The authors declare that they have no competing interests.
